# Customer discourse power and green innovation in industrial chain enterprises: A theoretical and empirical approach

**DOI:** 10.1371/journal.pone.0301598

**Published:** 2024-05-23

**Authors:** Yanwen Liu, Rui Wang

**Affiliations:** School of Economics and Management, Dalian University of Technology, Dalian, P. R. China; Inner Mongolia University, CHINA

## Abstract

Leading enterprises in the industry chain play a demonstrative role, and promoting green innovation among leading enterprises is a meaningful approach to unlocking industry chain dividends. According to an analysis of the game process between customers and leading firms that incorporates the open innovation theory, we find a nonlinear role for the consumer discourse power in the leading firms’ innovation. Furthermore, using data from Chinese A-share listed companies between 2012 and 2021, we observe an inverted "U" relationship between customer discourse power and green innovation. Notably, this effect is more pronounced in leading non-technology-intensive enterprises, industries with a high degree of industrial innovation, or regions with a high marketization degree. Our analysis also reveals that leading enterprises’ client leads to financial limitations that influence green innovation. Additionally, leading enterprises play a driving role in achieving "joint progress" in green innovation with local non-leading firms, and this effect exhibits spatial spillover.

## Introduction

Innovation serves as the engine of economic growth, and as green development becomes more refined, green innovation emerges as a critical driver of sustainable economic progress [1]. Compared to traditional innovation, green innovation, also known as "environmental innovation" and "eco-innovation," encompasses a range of green products and processes that improve or create designs, technologies, and products aimed at reducing or eliminating adverse environmental effects caused by resource use in enterprises. This type of innovation addresses stakeholders’ environmental concerns and facilitates the harmonious development of economic and environmental aspects among innovation subjects. As a significant proponent of ecological civilization, China has underscored the importance of high-quality growth, with innovation taking the lead and green practices assuming a dominant role. Enterprises choosing green innovation contribute significantly to reducing pollutant emissions and fostering environmental governance. As green innovation yields dual externalities of mitigating environmental damage and fostering knowledge creation, the traditional model of relying solely on internal resources for innovation development needs to be revised in facilitating green innovation activities. Enterprises must exchange resources with the outside world to effectively engage in green innovation to obtain essential innovation elements.

With the revolution in information technology and the deepening of the professional division of labor, innovation factors have demonstrated greater mobility and openness. On the one hand, fintech development has expanded access to credit for new and financially constrained borrowers, helping to ensure the continuity of innovation resources by alleviating firms’ capital constraints [2]. On the other hand, stock market connectivity improves the information efficiency of capital markets. It enables internal risk contagion and spillover effects, helping firms avoid risk factors and develop reliable innovation strategies [3]. However, in the context of the increasing resilience of the industrial chain supply chain, the vertical linkages of the industrial chain also realize the spillover and diffusion of innovation factors, and become an essential way for enterprises to obtain innovation resources [4]. Furthermore, stakeholder theory suggests that collaborative cooperation between firms and other innovation agents facilitates the realization of green innovation within the firm, with external stakeholders positively influencing corporate green innovation. Despite previous studies exploring the influence of external stakeholders, such as customer firms, on corporate green innovation [5,6], the effect and mechanism of customer discourse power on corporate green innovation have been overlooked. Customer discourse power represents a significant characteristic reflecting the relationship between customer firms and the supply chain. Specifically, the customer discourse power refers to the ability of the customer enterprise to dominate the business behavior in the process of trading with the supplier enterprise, which is mainly manifested by the bargaining power of the customer enterprise in the process of trading with the supplier. Relevant research is needed on the measurement method of customers’ voices. Still, the existing literature empirically tests that customer concentration affects the business behavior of upstream and downstream enterprises by affecting the bargaining power of both parties in transactions. Therefore, this paper uses customer concentration to measure customer voice power.

Furthermore, it is crucial to acknowledge that leading companies, acting as "precursor" and "bellwether" organizations, possess distinct advantages in terms of scale, technology, and market influence, thus exerting an impact on innovation through knowledge evaporation. On the one hand, drawing from agglomeration economy theory, leading firms exhibit a "lighthouse effect" on the aggregation of innovation factors. They foster technical information sharing through task division, resource sharing [7], and staff exchange [8]. On the other hand, leading industry chain firms occupy a core position in the innovation network. They augment the externalities of green innovation through localized competitive market incentives and technology diffusion, stimulating the innovation dynamics of other local firms and thereby impacting the overall innovation level of industry chain firms. Against this backdrop, several essential questions come to the forefront: Can customers influence the green innovation behavior of leading companies in the chain? If so, what is the specific mechanism of action? Does the spatial spillover of industrial chain leaders’ green innovation behavior exist? To address these questions, this paper examines the impact of customer discourse power on the green innovation of leading enterprises and its spatial spillover effect from the perspective of customer concentration. The study utilizes Chinese A-share listed companies as a sample, spanning the period from 2012 to 2021. Our goal is to investigate whether customers can contribute to the enhancement of green innovation among leading enterprises and explore the mechanisms through which customers affects the green innovation of these leading firms.

The potential contributions of this paper can be delineated in the following aspects: (1) Examination of Influencing Factors: This study delves into the influencing factors of green innovation in leading industry chain firms, focusing on exploring the relationship between customer companies and their green innovation. This research seamlessly integrates the perspectives of innovation, industry, and supply chains. As a result, it offers substantial empirical evidence concerning green innovation in leading firms, thereby significantly enriching the theoretical understanding of the influencing factors driving green innovation.

(2) Supply Chain Perspective: Adopting a comprehensive approach from the supply chain perspective, this paper adeptly explores the intricate interplay between customer discourse power and green innovation within leading enterprises operating within the industry chain. This examination effectively broadens the scope of existing research on supply chain relationships and the roles assumed by leading enterprises in the process.

(3) Spatial Durbin Model Analysis: To measure the spatial spillover effect of green innovation in leading enterprises, we use the spatial Durbin model as part of traditional econometric analysis. This innovative analytical framework provides an illuminating spatial explanation for the driving effect that leading enterprises exert within the industrial chain. Consequently, this valuable spatial insight is a crucial reference point for clarifying their instrumental role as "leading small enterprises with large ones."

## Literature review

### Stakeholders and corporate green innovation

First, we conducted keyword searches of the literature in Web of Science and CNKI (China National Knowledge Infrastructure), specifically, the following key terms were used: "innovation," "green innovation", "stakeholder", "interest subject", etc. The search of the literature revealed that Stakeholders can bring innovation-related resources such as capital, knowledge, and information to the firm and potentially be drivers of green innovation in the firm [9]. Studies have been conducted to examine the effect of stakeholders on corporate green innovation from several perspectives. Firstly, core stakeholders have a substantial stake in the firm and are direct drivers of corporate innovation. Employees are crucial human capital for enterprises and are crucial to product production and output innovation. Their environmental awareness and rational use of innovation resources can help enterprises achieve green innovation. As owners of social capital and information resources, shareholders can provide a professional perspective on corporate green innovation. Moreover, risk spillover, and abnormal volatility in stock prices will also have a significant impact on the development of innovation strategies, which offer insights into the financial limitations faced by leading enterprises and their potential impact on green innovation.

Secondly, dormant stakeholders will only actively interfere with corporate behavior and influence the legitimacy of business operations if their interests are unmet. This clear tendency becomes a "booster" for corporate green innovation. Among others, government departments can not only influence firms’ willingness to innovate by formulating environmental policies but can also use government subsidies and other means to motivate firms to produce more innovations [10]. Consumer demand and preference for environmentally friendly products continue to increase, and catering to this green preference has also become one of the driving forces for firms to engage in green innovation [9]. In addition, the superposition of competition and symbiosis among peer firms will bring about a cohort effect of innovation [11], increasing the overall level of green innovation in the industry.

### The impact associated with customer discourse power

According to stakeholder theory, large customers (higher customer concentration) play an essential role in the long-lasting trading relationship between trading parties and become an essential factor in determining the survival, operation, and development of a company. Therefore, customer concentration can also substantially impact a company’s production and operation. In recent years, scholars have conducted many valuable explorations of the impact effects of customer concentration. The two main literature areas closely related to this paper are customer concentration’s economic and non-economic effects on firms.

For one thing, there are economic-level effects of customer concentration on companies. It has shown that customer concentration can affect a firm’s asset utilization efficiency, M&A performance, corporate equity capital and capital market performance [12–15]. However, consistent conclusions on the economic consequences of customer concentration still need to be consistent. On the one hand, some scholars argued that a high level of customer concentration leads to inter-firm synergies, which not only help upstream and downstream firms in the supply chain to share resources and integrate technologies but also act as an informal external monitoring mechanism to improve the financial performance of firms. On the other hand, some scholars believe that when a firm faces a high concentration of customers, it is vulnerable to exploitation by large customers and secondary shocks from the business risks of the customer business. In this case, the company may adopt surplus management and other means to reduce business risk, but at the same time will damage the company’s financial performance.

Secondly, customer concentration can have a non-economic dimension on a business. With the growing emphasis on sustainable economic development in various countries, academics have begun to explore the effects of external business environments, such as customer concentration, on the non-economic aspects of firms. Developed from the perspectives of innovation resource crowding and individual customer characteristics, some scholars have empirically tested the inhibitory effect of customer concentration on firms’ technological innovation. However, some scholars have also used innovation knowledge sharing as an entry point to show that customer concentration can drive firms to achieve technological innovation [16].

In conclusion, a substantial body of research has examined how corporate green innovation and customer concentration affect businesses, confirming the beneficial benefits of customer concentration on business growth. However, there is still room for the current study to be expanded: (1) Stakeholder research on corporate green innovation needs to take consumer concentration into account when examining corporate green innovation. (2) With regard to the micro-impact effects of customer concentration, current research still needs to concentrate on the financial ramifications of customer concentration with a thorough examination of its impact and particular mechanisms of action in non-financial aspects, such as the green innovation of businesses. (3) Unlike other enterprises, leading companies have a specific voice in the industry. The micro-action mechanism of customer discourse power on their green innovation needs to be further explored. Therefore, the following research questions deserve to be explored: What is the effect of customers on green innovation of firms? Is there a spatial spillover effect of green innovation of leading firms?

## Model construction and research assumptions

### Problem description and model assumptions

Industry chain leading and customer enterprises are in the same industry chain, bringing resource spillover through transactions. However, due to the information asymmetry between the two parties in the initial stage, it is not possible to fully grasp the decision-making preferences of the two parties. There are differences in judgment and decision-making ability between the two parties, and the enterprises make the behavioral decisions most in line with the current environment through repeated learning and strategic adjustments. Therefore, this paper considers how customer enterprises influence the green innovation of leading enterprises through decision-making model construction.

#### Game analysis

First, the enterprise has two choices of innovative behavior: not carrying out green innovation and still producing products in the original technology field. Although this behavior is not in line with the deepening green consumption concept of consumers, it does not significantly increase the R&D cost for enterprises. Secondly, to carry out green innovation, the leading enterprises in the industry chain will actively carry out green innovation to maintain the industry chain status and continue to obtain monopoly profits. Simultaneously, they also face higher R&D costs. Secondly, customer enterprises also face two strategic choices: continuous cooperation and termination. Accordingly, this paper gives payment matrix and equilibrium strategies.

**Assumption 1** The utility *U* of the client firm is determined by the amount *S* of the transaction contract and the degree of product innovation. Product innovation is *P* when the product maintains the original innovation level, and product innovation is *P*_1_ when the green innovation technology is utilized for production.

**Assumption 2** The utility *V* of the leading enterprise of the industrial chain is determined by the green innovation R&D cost *C*. The enterprise’s product sales revenue *R*. *R*_1_ is the product revenue generated by the continuous cooperation of the existing client enterprise, and *R*_2_ is the enterprise’s sales revenue after the existing client enterprise stops the cooperation.

In summary, the payment matrix between customer enterprises and industry chain leading enterprises is shown in Table 1.

**Table 1:** Payment matrix.

customer enterprises	industry chain leading enterprises	
	green innovation	No green innovation
collaborative	[*U*_1_(*S*_1_, *P*_1_), *V*_1_(*R*_1_, *C*)]	[*U*_2_ (*S*_1_, *P*), *V*_2_(*R*_1_, 0)]
Termination of cooperation	[*U*_1_(*S*_1_, *P*_1_), *V*_1_(*R*_2_, *C*)]	[*U*_2_ (*S*_1_, *P*), *V*_2_(*R*_2_, 0)]

*Table 1 shows the payment matrix between customer enterprises and industry chain leading enterprises.

From the strategic choices and corresponding payment matrices of customer enterprises and industry chain leading enterprises in Table 1, it can be seen that the Nash equilibrium of the game between the two is (continuous cooperation, green innovation) and (termination of cooperation, no green innovation), i.e., not to carry out green innovation is the preferred strategy of the industry chain leading enterprises. However, the strategy of (continuous cooperation and green innovation), which maximizes the utility or welfare of the whole society, is not realized and falls into the "Prisoner’s Dilemma". The reasons for this phenomenon may be (1) the "short-sightedness" of the leading enterprises themselves. Compared with the benefits of green innovation, green innovation is characterized by high risk and high investment. Driven by the motive of "short-sightedness," leading enterprises may still choose not to carry out green innovation to maintain the enterprise’s financial status. (2) Compared with the information cost of changing suppliers, customer enterprises are more likely to maintain the existing industrial chain cooperative relationship. However, as the process from green patent application to authorization takes a long time, it takes several times for the customer and industry chain leading firms to complete the gaming process.

Accordingly, we argue that customer enterprises can influence the green innovation of leading enterprises in the transaction process, so this paper focuses on analyzing how customer enterprises and customer discourse power affect the green innovation of leading enterprises under the long-term cooperation relationship.

(2) Model Assumptions

Further analyzing the influence effect of customer enterprises on the green innovation of leading enterprises not only helps to improve the overall stability of the industrial chain but also helps to clarify the micro-action mechanism of external stakeholders on the green innovation of enterprises. Therefore, combining the above evolutionary game analysis process based on the open innovation theory, this paper puts forward the following hypotheses:

**Assumption 1** we assume that consumers are homogeneous and have separable preferences, and the effect function about the product is

$$\mu (q_{j})=\alpha q_{j}+\frac{1}{2}\beta q_{j}^{2}$$

Where *α*>0, *β*>0, *q*_j_ is the demand of the product consumers, assuming that the consumer’s expenditure on purchasing the product is *W*, then when the consumer utility is maximized:

$$\underset{q_{j}\geq 0}{\text{max}}\;\int_{0}^{\theta }\mu_{j}(q_{j})d_{j}s.t.\int_{0}^{\theta }\mu_{j}(q_{j})d_{j}=W$$

In order to solve the optimization, this paper obtains the inverse residual demand function about product *j*:

$$p_{j}(q_{j})=\frac{\mu \prime (q_{j})}{\mu }=\frac{\alpha -\beta q_{j}}{\mu }$$

**Assumption 2** Assume that the fixed cost of production of the leading firm is *c*_*f*_ and the marginal cost is ${\bar{c}}_{j}$, and when the firm chooses the optimal output level *q* (${\bar{c}}_{j}$, *μ*), the maximized profit is:

$$\underset{q_{j}\geq 0}{\text{max}}\;\pi (\bar{c_{j}};\mu )=\underset{q_{j}\geq 0}{\text{max}}\;p_{j}(q_{j})q_{j}-\bar{c_{j}}q_{j}-c_{f}$$

The optimal level is known by making dπ/d*q*_*j*_ = 0:

$$q(c_{j};\mu )=\frac{\alpha -c_{j}\mu }{2\beta }$$

Given this, there must exist *c*_*f*_ < *α*/*μ*, otherwise the firm will choose not to produce. And, according to the equilibrium output condition, it can be known that the maximized profit of the enterprise in the equilibrium state is:

$$\pi (\bar{c_{j}};\mu )=\frac{{\left(\alpha -\bar{c_{j}}\mu \right)}^{2}-4\beta \mu c_{f}}{4\beta \mu }$$

**Assumption 3** The original marginal cost of the enterprise is *c*_*j*0_, and with green innovation, the enterprise can reduce the marginal cost of its own production *c*_*j*_

**Assumption 4** Due to the characteristics of green innovation, the leading enterprise green innovation not only relies on the enterprise’s internal resources, but also will be affected by the enterprise’s external resource acquisition ability *δ*.

**Assumption 5** Assuming that the level of green innovation of the enterprise is *k*, the green innovation cost of the enterprise is a quadratic function about *k*, i.e.:

$$c_{k}=\varphi k_{j}+\frac{1}{2}\varphi k_{j}^{2}$$

### Model construction

(1) Green innovation decision of leading enterprises

According to the above assumptions can be obtained, the marginal cost of the enterprise is:

$$c_{j}=c_{j0}-\delta k_{j}$$

Combined with Assumption 5, the maximized profit of the leading enterprise is recalculated after including the leading enterprise’s green innovation cost in the enterprise’s revenue as:

$$\prod (c_{j0},k_{j};\delta ,\mu )=\pi (c_{j0}-\delta k_{j};\mu )-c_{k}-c_{f}$$

In equilibrium, the optimal green innovation level *k* of the enterprise satisfies the following first-order condition:

$$\delta q(c_{j0},k_{j};\delta ,\mu )=\varphi +\varphi k_{j}$$

At this time, the optimal output level is:

$$q(c_{j0},k_{j};\delta ,\mu )=q(c_{j0}-\delta k_{j};\mu )=\frac{\alpha -\mu (c_{j0}-\delta k_{j})}{2\beta }$$

In addition, assuming that the enterprise chooses the optimal green innovation level of k exists so that the slope of the marginal cost is strictly greater than the marginal benefit, at this point, the optimal green innovation level *k* can be obtained:

$$k_{j}=\frac{\alpha \delta -2\beta \varphi -\delta \mu c_{j0}}{2\beta \varphi -\delta^{2}\mu }$$

### Research hypothesis

Open innovation is a concept first proposed by Chesbrough, who pointed out that open innovation is distributed innovation that crosses organizational boundaries and manages knowledge flows effectively. Moreover, unlike closed innovation, open innovation theory emphasizes the need for firms to use both internal and external innovation resources and commercialization channels. In the context of open innovation, customer firms are the critical factors for upstream firms to carry out green innovation. Considering the power of customer enterprise discourse in the process of green innovation can not only reduce the enterprise’s new product development costs and risks but also help the upstream enterprise know the latest green consumption concepts of consumers, thus promoting the enterprise to produce personalized products to meet the consumers’ green needs, and ultimately achieve industrial chain synergy and new product sales performance enhancement. Research hypothesis are as follows:

(1) Analysis of the impact of customer discourse power on green innovation in leading firms. It has been shown that good innovation capability is an essential guarantee for firms to carry out continuous and stable innovation activities [17]. Therefore, we argue that customer discourse power may affect corporate green innovation by influencing the green innovation capabilities of leading firms. At the stage of relationship cultivation between enterprises and customers, to satisfy their demand for products and protect their relationship-specific investments, the green demands of customer enterprises can be transmitted to upstream enterprises through good interaction between the two sides of the transaction. Through the "green bullwhip effect", upstream enterprises are urged to conduct green innovation, research, and development. Moreover, when other companies in the industry improve their market position through green practices, the leading companies will strengthen their green innovation activities to reinforce their competitive advantages and consolidate their central position in the market. As a result, the information transfer between the two sides of the transaction is more efficient [18].

However, as customer-specific investments continue to increase and orders are concentrated in the hands of a few large customers, the bargaining power of customer firms increases. At this point, large customers have the motivation and power to monitor and certify their suppliers. Large customer firms not only demand that their supplier firms lower the price of their products and extend their credit terms but may also invest more in specialized assets to increase the firm’s financing constraints. The primary customer firms will not only demand that their supplier firms reduce product prices to extend credit terms but may also invest in more specialized assets to increase their financing constraints. Moreover, the external and high-risk nature of green innovation requires firms to access external resources such as capital and knowledge for substantial innovation [4] (Johnstone et al., 2009). However, excessive specialized investment and financing constraints will lead to insufficient resources for green innovation, hindering enterprise R&D and innovation. Therefore, client firms will increase the level of financing constraints faced by firms through the industrial chain discourse advantage, inhibiting leading firms from engaging in green innovation. Therefore, according to the above theoretical analysis, combined with the previous model of corporate innovation decision making, customer voice will be obtained by taking partial derivatives of μ, δ respectively:

$$\frac{\partial k_{j}}{\partial \delta }=\frac{2\beta \varphi (\alpha -\delta c_{j0}-\delta \mu )}{{\left(2\beta \varphi -\delta^{2}\mu \right)}^{2}}$$ (1)

$$\frac{\partial k_{j}}{\partial \mu }=\frac{\delta (\alpha \delta^{2}-2\beta \varphi c_{j0}-2\beta \varphi \delta )}{{\left(2\beta \varphi -\delta^{2}\mu \right)}^{2}}$$ (2)

Eqs (1) and (2) should be satisfied

$$\frac{\partial k_{j}}{\partial \delta }>0,\frac{\partial k_{j}}{\partial \mu }<0$$

Accordingly, we propose the following research hypotheses.

**Hypothesis 1**. There is an inverted "U-shaped" relationship between customer discourse power and green innovation in leading companies. That is, moderate customer discourse power helps leading firms to engage in green innovation, but too high customer discourse power will inhibit their green innovation.

#### "Winner-take-all" or "progress together"

The impact of green innovation by leading enterprises on non-leading enterprises. Driven by the accumulation forces of the localized environment, the leading firms will establish various linkages with other local firms so that other local firms will also be affected by the externalities of the leading firms. The existing literature shows that the management bias of the management is one of the important factors in determining the performance of smes in the face of Unexpected Shocks [19] (Zhou et al., 2022). This research dialectically analyses the effect of leading firms on the green innovation of other local firms from the perspective of industrial accumulation and market competition.

From the standpoint of industrial agglomeration, leading firms can drive the green innovation of other local firms through industrial linkages and technological spillover effects. Leading businesses typically have supply chains and industrial chains that are fairly thorough and entrenched locally. The presence of leading firms will enhance the interaction and matching companies and their clients, expand the green innovation resources of firms, and raise the overall level of green innovation in other companies. Leading enterprises are typically in the hub of the innovation cluster network in terms of technological spillover effects. Together with other local firms, they form an efficient and synergistic local innovation ecology, which can drive local non-leading enterprises to improve their efficiency and promote green innovation through knowledge spillover effects. However, from the perspective of market competition, green innovation represented by green products and processes not only reduces negative environmental externalities but also enhances the competitive advantage of firms [20], creating a "siphon effect" of green innovation by leading firms. Under this scenario, as local innovation resources are minimal, local non-leading firms will be more disadvantaged in the competitive market for innovation resources such as capital and technology and need to pay higher costs to obtain innovation factors.

At the same time, leading businesses will also use technical innovation resources to protect themselves from rivals and new entrants and to maintain their market share, which will ultimately drive up the cost of green innovation for regional non-leading businesses. Therefore, leading businesses may either play a "squeezing function" in deterring local non-leading businesses from pursuing green innovation or a "synergistic role" in fostering green innovation among local non-leading businesses. Accordingly, this paper proposes the following two competing hypotheses.

**Hypothesis 2a**. Green innovation by leading enterprises helps improve local non-leading firms’ green innovation level.
**Hypothesis 2b**. Green innovation by leading enterprises hinders the improvement of green innovation by local non-leading firms.

#### Spatial spillover effects of green innovation by leading enterprises

According to the new economic geography theory, factor flows, technological diffusion, and its spillover lead to increased spatial dependence between individuals. Spatial spillovers have been empirically tested in studies at the macro, medium (), and micro levels to determine whether they are crucial aspect of innovation activities [21]. From the perspective of green innovation in leading companies, firstly, as China’s marketization process advances, barriers to cooperation and investment between companies are gradually being broken down. Moreover, the "customer-supplier" relationship-based transactions are driving the introduction of technology between companies in different regions, enhancing the spatial links between firms in different regions regarding green innovation. Secondly, firms are not independent individuals from each other, variables like proximity to other businesses can cause agglomeration and diffusion effects of their innovative activities. Firms in various geographical regions drive firms in their own and neighboring regions to improve their green innovation output through regional flows of resources, imitation and learning, and collaborative innovation. Finally, the efficiency of information dissemination decreases as the distance grows, impediments to human mobility rise [22] (Eriksson and Lindgren, 2008), and the cost of cross-regional collaboration for businesses rises. As a result, the spatial spillover effect of green innovation diminishes with increasing spatial distance. In conclusion, this paper proposes the following research hypothesis.

**Hypothesis 3**. There is a spillover effect and spatial relevance to green innovation by leading enterprises.

**Fig 1:**
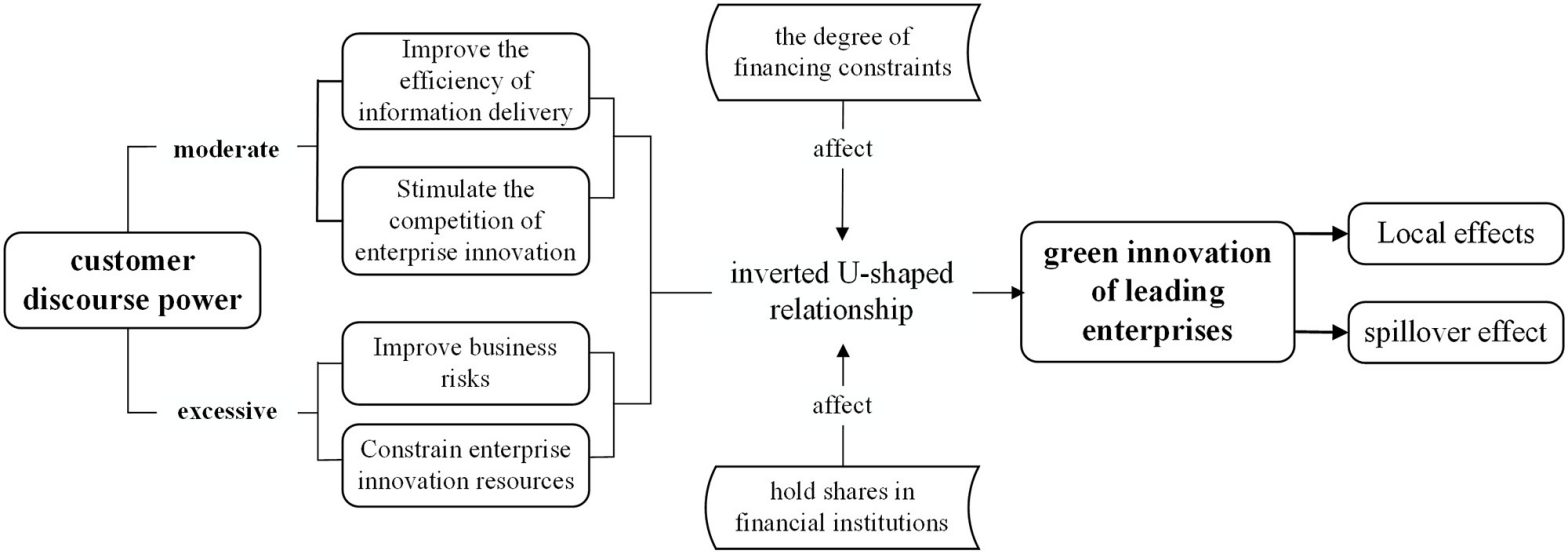
Theoretical analysis framework of customer discourse power of leading industry chain companies on their green innovation. *Fig 1 shows the logical framework of the theoretical analysis of this paper, which clearly shows the impact of moderate customer voice power and excessive customer voice power on the green innovation of leading enterprises, as well as the specific analysis of the spillover effect of green innovation in this paper.

The logical framework diagram of the research hypothesis is shown in Fig 1.

## Research design

### Sample selection and data sources

In this study, the research sample is made up of A-share listed companies in Shanghai and Shenzhen from 2012 to 2021. The sample is treated in accordance with the following rules: (1) Since the operating conditions of the sample enterprises treated by ST, *ST and other special treatments are not good, and the enterprises’ primary task is to maintain the enterprise operation rather than to carry out green innovations, we exclude the samples treated by ST, *ST and other special treatments;; (2) since the rules for preparing financial statements of enterprises in the finance and insurance industries are different from those of other entities, and since the assets of financial enterprises are mostly financial assets, the criteria for defining innovation capability are different from those of other industries, the sample of enterprises in the finance and insurance industries is excluded from the study; (3) Samples listed for less than one year are excluded due to the lack of data comparison of previous years at the early stage of listing and the significant fluctuation of their stock prices, so this part of the sample is excluded; (4) exclude samples with missing or abnormal data on critical variables. After the above treatment, the final sample of 9635 company-annual observations was obtained. Moreover, to minimize the impact of extreme values on the regression results, all continuous variables were subjected to a top and bottom 1% tail-shrinking process in this paper.

The data sources are as follows: corporate green patent data from the State Intellectual Property Office. The number of benefit applications is a public database and updated weekly to help grasp the latest environmental technology trends of leading enterprises. Corporate latitude and longitude data and corporate financial data from the China Stock Market and Accounting Research (CSMAR) databases, and geographical green patent innovation from the China Research Database Services (CNRDS) database. The innovation of regional green patents was derived from the CNRDS database, customer discourse power was derived from the China National Research Data Service (CNRDS) database, green patent data of leading enterprises were obtained from the State Intellectual Property Office, enterprise latitude and longitude data and enterprise financial data were obtained from the CSMAR database.

### Variable definition and calculation

#### Explained variable

Leading enterprises’ green innovation level. In particular, for the measurement of leading firms, this paper uses the measure of Jannati [8] (Jannati, 2020) to identify the size of companies as leading enterprises. Specifically, this paper defines the top 50 enterprises in each two-digit industry in terms of total assets for the year as the leading firms. It organizes them according to their industries and cities to derive data on the leading enterprises in each industry nationwide. For the measurement of green innovation, we draw on Hao and He [23] (Hao and He, 2022), using the total number of green patent applications by enterprises to measure the level of green innovation, which is calculated by adding one to the number of green patent applications by firms and taking the natural logarithm. The reason for choosing this measurement is that the green patent application data covers multi-dimensional information such as technology category and application time, which provides a reliable data guarantee for an in-depth study of enterprise green innovation. Therefore, we utilize green patent application data to measure the green innovation of leading enterprises.

#### Explanatory variable

Customer discourse power. Customer discourse power reveals how reliant suppliers are on their clients. According to Cao et al.[24] (Cao et al., 2021), We use customer concentration as a proxy for customer discourse power, specifically, we measure customer concentration using the combined sales of the top five clients by the total yearly sales reported in the annual report.

#### Control variables

To control for other characteristic indicators that affect corporate green innovation, this paper refers to the studies of Liu et al [25], and Hao and He [23]. We select the following control variables: Firm size (*Size*): We use natural logarithm of the annual assets to measure firm size. Financial leverage (*LEV*): The level of financial leverage will also affect the green innovation of enterprises. When the financial leverage of enterprises is too high, the tendency of green innovation will be reduced. In our research, we use total liabilities /total assets to measure it. Profitability (*ROA*): Firms with higher profitability have a stronger tendency towards green innovation. We use net profit/total assets to measure it. Cash holding level (*Cash*): the cash holding level reflects the capital liquidity of enterprises, and Cash and cash equivalents/total assets is used to measure the cash holding level of enterprises. Proportion of fixed assets (*Fixed*): We use total fixed assets/total assets to measure it. Firm value (*Tobin’s* Q): we use enterprise Tobin Q value to measure it. Book-to-market ratio (*MB*): we use book value/total business market capitalization to measure it. Year of establishment (*lnage*): Firms in the growth and maturity stages may have greater green innovation potential, while firms in the decline stage have a lower propensity for green innovation. Institutional investor ownership (*Invest*): As important external shareholders of enterprises, institutional investors influence important strategic decisions of enterprises by virtue of their professionalism and information ability. Board size (*Board*): Board resolutions will have an impact on green innovation, and this paper uses the natural logarithm of the number of board members to measure the size of the board. The proportion of independent directors (*Indep*): we use The ratio of the number of independent directors to the number of board members to measure it. Dual role (*Dual*): The situation of dual employment will generate agency problems, which will have an impact on innovation strategy. If the chairman of the company is also the general manager, the value is 1, otherwise it is 0. In addition, we control for a time dummy variable (*Year*), an industry dummy variable (*Ind*) and a province dummy variable (*Pro*).

The major variables and data descriptions are displayed in Table 2.

**Table 2:** Description of the main variables.

Type	Symbol	Variable definition and calculation
Explained variable	*Grpas*	Natural logarithm of the total number of green patent applications filed by the enterprise in the current period (applications+1)
Explanatory variable	*Buycc*	Total sales of the top five customers/total sales for the whole year
Control variables	*Size*	Natural logarithm of the annual assets
	*LEV*	Total liabilities /total assets
	*ROA*	Net profit/total assets
	*Cash*	Cash and cash equivalents/total assets
	*Fixed*	Total fixed assets/total assets
	*Tobin’s Q*	Enterprise Tobin Q value
	*MB*	Book value/total business market capitalization
	*lnage*	Natural logarithm of the age of the enterprise(years+1)
	*Invest*	The percentage of shares held by institutional investors in the year
	*Board*	Natural logarithm of the number of board members
	*Indep*	The ratio of the number of independent directors to the number of board members
	*Dual*	Dummy variable: If the chairman of the company is also the general manager, the value is 1, otherwise it is 0
	*Year*	Year value
	*Ind*	Primary industry classification according to the standards of CSRC
	*Pro*	Province dummy variable

*Table 2 reports the definition and calculation of this paper’s main variables, including explained, explanatory, and control variables.

### Test model design


$$\begin{array}{ll} Grpas_{i,t} & =\alpha_{0}+\alpha Buycc_{i,t}+\alpha_{2}Controls_{i,t}+\sum Ind\\ & +\sum Year+\sum Province+\varepsilon_{i,t} \end{array}$$ (3)

Where *Grpas*_*i*,*t*_ is the level of green innovation of leading firm *i* in year *t*, *Buycc*_*i*,*t*_ is its customer discourse power in year *t*, and *Controls*_*i*,*t*_ is all the control variables in this paper. *Year*, *Ind*, and *Province* denote the year, industry, and province fixed effects of the controls. *ε*_*i*,*t*_ is the error term.

## Analysis of empirical results

### Descriptive statistical analysis

Descriptive statistics are provided for the key variables in Table 3. Among them, the mean value of total green patent applications (*Grpas*) of leading enterprises was 0.831, with a median of 0 and a standard deviation of 1.175, showing that the overall level of green innovation of leading enterprises in China is low and there are significant individual differences. The mean value of customer discourse power was 0.268, with a standard deviation of 0.216. In terms of the control variables, the median financial leverage of the sample enterprises was 48.7%, showing that the debt level of the sample enterprises was in a relatively reasonable range. Additionally, the statistical results of the other control variables were also within the normal range.

**Table 3:** Descriptive statistics of major variables.

Variables	N	mean	sd	min	p50	max
*Grpas*	9635	0.831	1.175	0.000	0.000	5.069
*Buycc*	9635	0.268	0.216	0.008	0.202	0.973
*Buycc2*	9635	0.119	0.186	0.000	0.041	0.947
*Size*	9635	22.957	1.340	20.375	22.850	26.724
*Lev*	9635	0.476	0.198	0.074	0.487	0.871
*ROA*	9635	0.046	0.041	0.001	0.034	0.193
*Cash*	9635	0.061	0.049	0.002	0.050	0.210
*Fixed*	9635	0.227	0.165	0.003	0.196	0.668
*Tobin’s Q*	9635	1.772	1.032	0.863	1.423	6.561
*MB*	9635	1.463	1.460	0.128	0.952	7.902
*lnAge*	9635	2.922	0.333	1.812	2.996	3.497
*Invest*	9635	0.463	0.228	0.009	0.483	0.900
*Board1*	9635	2.170	0.181	1.792	2.197	2.708
*Indep1*	9635	0.376	0.054	0.333	0.364	0.571
*Dual1*	9635	0.212	0.409	0.000	0.000	1.000

*Table 3 reports the descriptive statistics of the main variables. The table presents mean, median, standard deviation, and maximum for each variable. The sample period spans the period 2012 to 2021.

### Regression results and analysis

To test research Hypothesis 1, regression analysis was conducted using an OLS model; firm-level clustering robust standard errors were also used in the regressions to correct for possible estimation bias caused by heteroskedasticity. The regression results of model (3) are reported in Table 4. As can be seen, there is no linear correlation between customers and the green innovation of top enterprises. The regression results in column (1) include only the primary term of customer discourse power (*Buycc*), whose regression coefficient is insignificant. However, after adding the quadratic term of customer discourse power (*Buycc2*) in column (2), its regression coefficient is significant at the 5% level; at this point, the regression coefficient of the primary term of customer discourse power is significantly positive. This result indicates an inverted "U" shaped relationship between customers and green innovation of leading firms, research Hypothesis 1 was confirmed. Additionally, this study determines that the inflection point of the inverted "U" curve is equal to 0.443, indicating that customer discourse power aids in encouraging green innovation in leading businesses when it is lower than 44.3%. However, when customer discourse power is higher than 44.3%, customer discourse power hinders green innovation in leading firms. This outcome validates H1. A potential explanation for this particular outcome could be attributed to a finance constraint: On the one hand, in the early stage of establishing the transaction relationship, there is information asymmetry in the initial stage of the transaction, and the two parties need help to grasp the decision-making preferences of the other party fully. Moreover, there are differences in judgment ability, decision-making ability, and other aspects between the two sides of the transaction, which must be repeatedly learned and adjusted. On the other hand, with the continuous increase of customer-specific investment, corporate orders are gradually concentrated in the hands of a few large customers, and the bargaining power of customer firms also increases. As externalities and high risk characterize green innovation, more financial support is needed for substantive innovation. Excessive professional investment and financing constraints will lead to insufficient resources flowing to green innovation, hinder enterprise R&D and innovation, and then affect the green innovation of leading enterprises. A high level of financial constraints will lead to more resources flowing to green innovation, hindering enterprise R&D and innovation and ultimately reducing the green innovation of leading enterprises. Therefore, the voice of customers will impact the green innovation of leading enterprises by affecting the financial constraints and innovation motivation of supplier enterprises. The specific mechanism of action is shown in Section 5.1.

**Table 4:** Results of customer concentration on green innovation of leading enterprises.

Variables	(1)	(2)
	Grpas	Grpas
*Buycc*	0.047 (0.446)	0.663** (2.377)
*Buycc2*		-0.748** (-2.406)
*Size*	0.374*** (12.737)	0.378*** (12.916)
*Lev*	-0.055 (-0.423)	-0.050 (-0.388)
*ROA*	0.154 (0.576)	0.195 (0.731)
*Cash*	-0.353* (-1.769)	-0.326* (-1.651)
*Fixed*	-0.314** (-2.184)	-0.295** (-2.056)
*Tobin’s Q*	0.053*** (3.293)	0.054*** (3.380)
*MB*	0.015 (0.619)	0.015 (0.658)
*lnAge*	0.114 (1.352)	0.118 (1.410)
*Invest*	-0.083 (-1.021)	-0.075 (-0.918)
*Board*	0.438*** (2.956)	0.447*** (3.022)
*Indep*	0.817** (2.032)	0.835** (2.077)
*Dual*	0.032 (0.835)	0.032 (0.837)
*Year*/*Ind/Pro*	Yes	Yes
*Constant*	-9.347*** (-11.628)	-9.571*** (-12.053)
*N*	9635	9635
*R* ^*2*^	0.476	0.477

* Table 4 reports the analysis of regression results of Model (1) using the OLS model.

Note: ***, **, and * indicate significance at the 1%, 5%, and 10% levels, respectively. The same table below.

### Group tests

#### Grouping regression based on industry type

We use the classification to reveal the effect of customers on the industry heterogeneity of leading firms’ green innovation. According to the variability of the firms’ industries, we divide the sample firms into two sub-samples of technology-intensive industry companies (*TI* = 1) and non-technology-intensive industry companies (*TI* = 0) and then perform regression analysis. The regression results are reported in Table 5. According to the regression results in columns (1) and (2) of Table 5, customer concentration’s effect on green innovation in leading firms is more significant in non-technology-intensive firms. Specifically, the regression coefficient of concern in this paper is -0.746 in non-technology-intensive industries, which is significant at the 5% level. While the regression coefficient of *Buycc2* in technology-intensive industries is not significant. Both pass the between-groups difference test. This may be because, compared with non-technology-intensive enterprises, technology-intensive enterprises are the leaders in technological innovation; advanced technology is the critical resource that technology-intensive enterprises are constantly pursuing and indispensable. Technology-intensive enterprises face the development dilemma of rapid product replacement and must constantly innovate to expand market share. Moreover, technology-intensive enterprises need more funds in the innovation process, and the capital of upstream and downstream enterprises more significantly constrains them. Therefore, among the research results of this paper, the research results of technology-intensive enterprises are more significant.

**Table 5:** Regression results for a grouping test.

Variables	(1)		(2)		(3)	
	TI = 0	TI = 1	HHI = 0	HHI = 1	market = 0	market = 1
*Buycc*	0.571*	1.215**	0.359	1.316***	0.006	0.861**
	(1.831)	(2.123)	(1.110)	(3.165)	(1.564)	(2.260)
*Buycc2*	-0.746**	-1.083	-0.483	-1.475***	-0.000*	-0.782*
	(-2.183)	(-1.581)	(-1.357)	(-3.097)	(-1.710)	(-1.851)
*Controls*	Yes	Yes	Yes	Yes	Yes	Yes
*Year*/*Ind/Pro*	Yes	Yes	Yes	Yes	Yes	Yes
*Constant*	-8.800***	-10.559***	-8.253***	-11.110***	-8.482***	-10.542***
	(-9.959)	(-6.130)	(-8.811)	(-10.311)	(-7.654)	(-10.423)
*N*	7298	2337	3581	6054	4919	4716
*R* ^*2*^	0.469	0.510	0.476	0.502	0.488	0.482

*Table 5 reports the regression results of the grouping test in this paper. Columns (1) and (2) of Table 5 are the group regression based on industry type, columns (3) and (4) show the group test based on the industry competitive environment, and columns (5) and (6) show the impact effect of green innovation on customer concentration in different groups.

#### A group test based on the industry’s competitive environment

The competitive market environment can profoundly affect the innovation behavior of firms. In this paper, the Herfindahl Index (*HHI*) is used as a proxy variable to measure the competitive environment of an industry. This paper divides the sample firms into two sub-samples of the highly competitive environment (*HHI* = 1) and the lower competitive environment (*HHI* = 0), based on the median of the *HHI*, and performs group regressions. The estimation results are shown in columns (3) and (4) of Table 5. The regression results in column (3) show that the regression coefficient of *Buycc2* passes the 1% significance level test for firms in a lower competitive environment, implying that customers can significantly impact their green innovation. However, in column (4), the coefficient of *Buycc2* does not pass the significance level test, showing that customers do not significantly impact green innovation for firms in a highly competitive environment. It’s possible that the advantages of the leading enterprises are diminished when they are subject to greater industry competition. The buyer firms have more substantial bargaining power in the transaction process, which will increase the business risks of the seller firms and reduce their R&D investment by lowering the sales price and extending the credit period, ultimately discouraging them from green innovation.

As a result of the buyer firms’ stronger negotiating position during the transaction process, the seller firms’ business risks will rise, and by lowering the sales price and extending the credit period, they will invest less in R&D, ultimately discouraging them from making green innovations.

#### A group discussion of considering the marketization process

Marketization reform helps to improve market mechanisms and enrich factor markets, which in turn creates a more robust atmosphere for science and technology innovation and promotes firms to improve their innovation performance. Therefore, we use the marketization index to represent the marketization process of the region in which the companies are located. Specially, we divide the overall sample into two categories, high marketization (*market* = 1) and low marketization (*market* = 0), based on the median of the marketization index. The effects of customers in the various groups on green innovation are reported in columns (5) and (6) of Table 5, respectively. It can be seen that the coefficients of *Buycc2* pass the significance level test in both the samples from regions with high and low levels of marketization. Moreover, the coefficient of the effect of customers on the green innovation performance of the leading firms located at higher levels of marketization is -0.782, with a more considerable absolute value of the effect coefficient. This indicates that the inverted "U" relationship between customer voice and the innovation of leading enterprises is more pronounced in regions with a high level of marketization. The possible reason for this result is that the higher degree of marketization has a better-developed product market, factor market, and technology market, which can reflect the supply and demand relationship of innovation resources in a timelier manner, accelerate the transfer of innovation resources such as workforce, capital, and technology, and then have a more noticeable impact on the green innovation performance of leading firms.

### Endogenous testing

The relevant factors that may affect the green innovation of leading enterprises are controlled in the baseline regression model. However, problems like missing variables and measurement errors in variables could still occur. As a result, we address the potential endogenous issues from the following angles, and the results are shown in the Table 6.

**Table 6:** Results of customer concentration on green innovation of leading enterprises.

Variables	(1)	(2)	(3)	(4)	(5)
				*T*+1	*T*+2
	Grpas	Grpas	Grpas	Grpas1	Grpas2
*Buycc*	1.021*	0.403*	0.614**	0.644**	0.734**
	(1.668)	(1.688)	(2.182)	(2.155)	(2.327)
*Buycc2*	-0.980*	-0.464*	-0.714**	-0.659*	-0.740**
	(-1.797)	(-1.761)	(-2.272)	(-1.955)	(-2.035)
*Controls*	Yes	Yes	Yes	Yes	Yes
*Year*/*Ind/Pro*	Yes	Yes	Yes	Yes	Yes
*Constant*	-8.731***	-6.795***	-9.859***	-10.086***	-9.875***
	(-7.490)	(-9.675)	(-11.848)	(-11.962)	(-10.995)
*N*	3527	9635	9635	6611	5773
*R* ^*2*^	0.501	0.483	0.479	0.487	0.483

* Table 6 reports the endogeneity test of this paper. They are PSM, controlling individual fixed effects at the enterprise level, adding control variables, and changing time series.

#### Propensity Score Matching (PSM)

In order to overcome the sample selection bias, we divide the sample enterprises into high-concentration and low-customer-concentration groups according to the median customer concentration. Then, we use the propensity score matching method to regress the sample enterprises. First, relevant business variables such as enterprise size, debt level, profitability, and firm value are selected as covariates, and the propensity score is calculated using the Logit model. Secondly, according to the one-to-one principle, the nearest neighbor matching method selects matching samples. The matched results pass the balance test.

#### Control the individual fixed effects at the firm level

We use the firm fixed effect model for re-estimation to control the individual-level influence factors that do not change over time. The regression results are displayed in Table 6’s column (2). The regression results support the previous research conclusions.

#### Increase the control variable

We add potential missing variables such as corporate profitability, the share of the largest shareholder, and environmental regulations. The conclusion of the study is consistent with the previous results.

#### Change the time series

Since green innovation has the characteristics of high risk and slow return, substantial green innovation usually takes a long time. Therefore, we use the number of green patent applications of leading enterprises in the *t*+1 and *t*+2 years to measure the level of green innovation. Then makes a regression again. The specific regression results are shown in columns (4) and (5) of Table 6, consistent with the previous baseline regression results.

### Robustness tests

#### Changing the explanatory variables

The number of green utility patent applications was used as the explanatory variable, and a re-regression analysis was conducted in Table 7. At this point, this paper’s regression coefficient of interest was negative at the 1% significance level, consistent with the previous findings.

**Table 7:** Robustness test regression results.

Variables	(1)	(2)	(3)
	*Grpas-re*	*Grpas*	*Grpas*
*Buycc*	0.957***	0.663***	0.883***
	(3.146)	(4.167)	(2.874)
*Buycc2*	-1.056***	-0.748***	-1.036***
	(-2.952)	(-4.118)	(-3.023)
*Controls*	Yes	Yes	Yes
*Year*/*Ind/Pro*	Yes	Yes	Yes
*Constant*	-8.137***	-9.161***	-8.973***
	(-8.846)	(-23.533)	(-10.232)
*var*		0.491***	
		(61.685)	
*N*	9635	9635	4776
*R* ^*2*^	0.507	/	0.457

* Table 7 reports the robustness test results of this paper, including changing the explained variable, changing the measurement model, and re-selecting the sample regression window.

#### Replacement of the econometric model

Since green innovation of leading firms is a discrete value with 0 as the lower bound, the explanatory variables in this paper are still typical left-truncated tail data. Therefore, we use the Tobit model to re-run the regression of Eq (1). The test results are shown in column (2) of Table 7, and the findings remain unchanged.

#### Re-selection of the sample regression window

China’s index of green patent applications has changed since 2017. As a result, the samples from 2018 and later were not included in this study, and the sample data from 2012 to 2017 were re-regressed. The regression results are shown in column (3) of Table 7, and the results are consistent with the previous baseline regression results.

The results of the above robustness tests are consistent with the results of the benchmark regressions, supporting the conclusion that there is an inverted "U-shaped " relationship between customer discourse power and green innovation of leading firms, indicating that the results of the previous benchmark regressions are robust.

## Discussion

### Analysis of the mechanism

The theoretical analysis above points out that customers can affect the green innovation behavior of leading enterprises by enhancing their financing constraints. Therefore, in order to verify the role mechanism of financing constraints in the relationship between customer discourse power and green innovation of leading enterprises, this paper constructs a regression model as follows:

$$\begin{array}{c} Median_{i,t}=\theta_{0}+\theta_{1}Buycc_{i,t}+\lambda Controls_{i,t}\\ +\sum Ind+\sum Year+\sum Province+\varepsilon_{i,t} \end{array}$$ (4)

#### Financing constraints

According to the previous theoretical analysis, customers will act on green innovation by affecting the financing constraints of leading firms. To verify the existence of this mechanism of action, we refer to Kaplan and Zingales (1997) and select the KZ index as a proxy variable for firms’ financing constraints. The more extensive the KZ index, the stronger the financing constraints faced by the sample firms. The specific measurement of the KZ index is as follows.

$$KZ_{i,t}=\beta_{1}X_{1it}+\beta_{2}X_{2it}+\beta_{3}X_{3it}+\beta_{4}X_{4it}+\beta_{5}X_{5it}$$ (5)

Where *X*_*1it*_, *X*_*2it*_, *X*_*3it*_, *X*_*4it*_, and *X*_*5it*_ are net cash flow from operations/total corporate assets, dividend payout ratio, ratio of corporate cash holdings to total corporate assets, gearing ratio, and corporate Tobin’s Q value, respectively.

The specific regression results are presented in column (1) of Table 8. As can be seen, the regression coefficient of interest is 0.080, it passes the 5% level of a significance test, suggesting that higher customer discourse power can influence the green innovation of leading firms through financing constraints. This regression result further validates the research Hypothesis 1.

**Table 8:** Mechanism test regression results.

Variables	(1)	(2)	(3)	(4)
	KZ	CFIN = 1	CFIN = 0	Erisk
*Buycc*	-0.051	0.576	0.713*	0.007
	(-1.633)	(1.553)	(1.956)	(0.805)
*Buycc2*	0.080**	-1.053**	-0.344	-0.002
	(2.399)	(-2.441)	(-0.897)	(-0.165)
*Controls*	Yes	Yes	Yes	Yes
*Year*/*Ind/Pro*	Yes	Yes	Yes	Yes
*Constant*	-3.451***	-9.134***	-8.298***	0.074***
	(-30.593)	(-8.962)	(-7.107)	(3.740)
*N*	9635	5731	3904	9635
*R* ^*2*^	0.909	0.513	0.499	0.236

* Table 8 reports the action mechanism in the relationship between customer concentration and green innovation of leading enterprises. In this Column, (1) is the test result of financing constraints, columns (2) and (3) are the hedging effect of enterprises’ equity participation in financial institutions, and Column (4) is the test result of enterprises’ innovation motivation, which supports the previous hypothesis H1.

#### The hedging role of corporate equity participation in financial institutions

It has been shown that the advantages of capital, credit, market, and other factors that financial institutions have can effectively alleviate the financing constraints faced by enterprises. Therefore, if higher customer discourse power weakens a firm’s ability to innovate green by raising its level of financing constraints, then the negative impact of financing constraints will be significantly reduced when alternative financing channels exist. Therefore, we use the data of "corporate equity participation and shareholding companies" in the CNRDS database to measure the specific situation of corporate equity participation and shareholding in financial institutions. Specifically, this paper uses the dummy variable *CFIN* to measure the specific case of corporate shareholdings in financial institutions, with *CFIN* taking a value of 1 if the company holds an equity stake in the financial institution in the current year and 0 otherwise. The specific regression results are shown in columns (2) and (3) of Table 8. This result shows that the regression coefficients of interest in this paper are significantly negative when leading firms hold equity in financial institutions and pass the 5% significance level test. This indicates that when a firm faces high customer concentration, if it has equity in a financial institution, it can counteract the adverse shocks in financing caused by customer concentration, further supporting the previous research Hypothesis 1.

#### Motivation to innovate

Since green innovation activities are characterized by high risk and high investment, when firms face operational risks arising from higher customer discourse power, management will reduce operational risks by discouraging firms from engaging in green innovation activities. Because of this, we use corporate earnings volatility to measure the level of risk-taking (*Erisk*) of firms [26] and uses it as a proxy variable for regression analysis.

The regression result shows that the regression coefficient of *Buycc2* does not pass the significance level test. Customers does not affect green innovation in leading firms by weakening their incentive to innovate. In summary, increasing firms’ financing constraints is the primary channel through which customer discourse power changes leading to firms’ green innovation level. This finding supports the previous research Hypothesis 1.

### The "radiating" effect on non-leading enterprises

It has been shown that although regressions can be directly validated using the level of green innovation of leading local firms versus non-leading firms, such studies suffer from serious endogeneity problems. Therefore, we construct the following regression model using national *M&A* policy as an exogenous shock [27].


$$\begin{array}{ll} NLFGrpas_{i,t} & =\varphi_{0}+\varphi_{1}LFGrpas+\varphi_{2}LFGrpas\times MAply+\varphi_{3}Controls_{i,t}\\ & +\sum Ind+\sum Year+\sum Province+\varepsilon_{i,t} \end{array}$$ (6)

*MAply* is the M&A policy; if the industry in which the firm is located is one of the industries supported by the National Five-Year Plan of China, then the value is 1; otherwise, it is 0. Additionally, we measure the firm’s level of green innovation using the natural logarithm of the number of green patent applications in the year + 1. Moreover, the natural logarithm of the number of green patent applications in the year + 1 is used to measure enterprises’ level of green innovation in the regression process. Table 9 reports the specific regression results for model (6).

**Table 9:** Results of regression of local non-leading enterprises.

Variables	(1)	(2)
	coefficients	T value
*LFGrpas*	0.041**	(2.472)
LFGrpas*MAply	0.001***	(3.136)
*MAply*	0.061	(1.341)
*Controls*	Yes	
*Year*/*Ind/Pro*	Yes	
*Constant*	7.358***	(15.116)
*N*	9635	
*R* ^*2*^	0.907	

* Table 9 reports Model (5) regression results, which indicate the spatial spillover effect of green innovation among cities associated with corporate green innovation.

The regression results show that the regression coefficient of *LFGrpas* × *MAply* is 0.001, passing the 1% significance test. It suggests that those of leading firms may influence local non-leading enterprises’ levels of green innovation, and this result supports the previous research Hypothesis 2a. The reasons for this may be that, firstly, the existence of industry chain leaders improves the connection and matching between clients and suppliers, increasing their green innovation resources and enhancing those of other businesses. Secondly, local enterprises collaborate to establish a productive and synergistic local innovation ecosystem, which enhances the knowledge-spillover impact of invention and encourages local non-leading businesses to pursue green innovation.

### Spatial autocorrelation test

#### Spatial autocorrelation test and model setting

Initially, scholars judged whether innovation was spatially relevant from whether the borders of two regions bordered each other. As spatial econometric research developed, pertinent elements like geographic distance were increasingly added to the spatial spillover model’s logical structure. So, we use the spatial neighborhood (0–1) matrix and the economic geography matrix to conduct spatial econometric regressions. Meanwhile, in order to explore the spatial spillover effect of green innovation of leading enterprises, a spatial Durbin model (SDM) was constructed. The specific formula is as follows.

Meanwhile, a spatial Durbin model (SDM) was built to investigate the spatial spillover effect of top businesses’ green innovation.


$$\begin{array}{c} CityGrpas_{i,t}=\eta_{0}+\rho \sum jW_{ij}CityGrpas_{i,t}+\eta_{1}LFGrpas_{i,t}+\eta_{2}NLFGrpas_{i,t}\\ +\eta_{3}\sum jW_{ij}LFGrpas_{i,t}+\eta_{4}\sum jW_{ij}NLFGrpas_{i,t}+\eta_{5}Controls_{i,t}+\varepsilon_{i,t} \end{array}$$ (7)

Where *CityGrpas* is the level of green innovation in neighboring regions, *LFGrpas* is the level of green innovation in leading local firms, and *NLFGrpas* is the level of green innovation in non-leading local firms.

#### Spatial autocorrelation test of green innovation of leading enterprises

Before using the spatial econometric model to calculate the spillover effect of leading enterprises’ innovation, enterprises’ green innovation level should be tested for spatial correlation. In relevant studies, the spatial adjacency and economic geography matrices are mainly used to measure the spatial spillover effect. Specifically, in the spatial adjacency matrix, the value between adjacent prefecture-level cities is 1, and the value is 0 if they are not adjacent. The economic distance matrix introduces the difference in per capita GDP of the prefecture-level city where the enterprise is located as an index to measure the economic distance of the prefecture-level city, and the spatial weight matrix can better conform to the development of the regional economy in China. Therefore, we measure the global and local Moran indices of green innovation of leading enterprises based on the spatial neighborhood (0–1) matrix and the geographical distance weight matrix. Overall, the Moran indices are above 0.1 and pass the significance level test. This indicates a positive spatial spillover effect of green innovation of leading enterprises.

#### Spatial spillover effects of green innovation by leading firms

Table 10 reports the regression results of model (7).

**Table 10:** Regression results for spatial spillovers.

Variables	(1)		(2)	
	neighborhood matrix		the geographical distance weight matrix.	
*LFGrpas*	0.200***	(43.97)	0.169***	(40.75)
*NLFGrpas*	0.698***	(156.40)	0.775***	(194.13)
*Buycc*	0.0161	(0.90)	0.00572	(0.38)
W*LFGrpas	-0.123***	(-10.97)	-0.208***	(-17.20)
W*NLFGrpas	-0.233***	(-9.88)	-0.987***	(-81.30)
W*Buycc	0.0405	(0.43)	0.126	(1.78)
*ρ*	0.470***	(19.00)	1.186***	(165.81)
*Year/Space*	Yes		Yes	
*N*	5944		5944	

* Table 10 reports the results of the effect decomposition of the SDM model. It is decomposed into direct effect, indirect effect, and total effect.

Note: Spatial regression results for control variables are not shown in the table, the same as the table below. The change in the observed values is due to the balance panel data.

It can be seen that the coefficients *ρ* of the spatial lags of both spatial matrices are significantly positive, indicating that there is a spatial spillover effect of green innovation among cities associated with corporate green innovation. This implies that leading firms that engage in green innovation will not only drive up the level of green innovation in their regions but will also drive companies in neighboring regions to improve their green innovation performance through the spatial spillover effect.

#### Decomposition of the spatial effects of green innovation in leading enterprises

We use the spatial Durbin model to measure the spatial spillover effect, and because the explanatory variables in the spatial Durbin model include the deformation of the explained variables, which violates the assumption that the explanatory variables in the traditional spatial measurement model are strictly exogenous, the coefficient estimates of the spatial Durbin model are different from those of other spatial measurement models. As a result, the impact of explanatory variables on explained variables cannot be fully reflected. A study shows that the direct regression results of the SDM model cannot accurately represent the degree of influence between variables when the coefficients of the spatial lags of the explanatory variables are significantly non-zero. Therefore, the results of the SDM model should be decomposed into direct, indirect, and total effects using partial differential methods. The direct effect is the effect of the leading enterprises in the region on the green innovation of local enterprises, the indirect effect is the promotion effect of the green creation of the leading enterprises in the area on the green innovation of enterprises in other regions, and the total product is the sum of the direct impact and the indirect effect. Table 11 reports the decomposition results of the effects of the SDM model.

**Table 11:** Effect decomposition of the spatial Durbin model.

Variables	(1)			(2)		
	Direct	Indirect	Total	Direct	Indirect	Total
*LFGrpas*	0.198***	-0.0544**	0.143***	0.168***	0.0431	0.211***
	(43.42)	(-2.95)	(7.65)	(38.51)	(0.72)	(3.60)
*NLFGrpas*	0.705***	0.173***	0.878***	0.766***	0.369***	1.135***
	(166.30)	(11.98)	(60.14)	(182.03)	(6.99)	(21.59)
*Buycc*	0.0211	0.0757	0.0969	0.0244	-0.723	-0.698
	(1.18)	(0.44)	(0.54)	(1.26)	(-1.73)	(-1.73)

Firstly, in the direct effect, the green innovation of the leading enterprises has a significant positive impact on the green creation of local enterprises, indicating that the top enterprises can achieve the spillover effect of invention through industrial agglomeration and knowledge sharing. Secondly, in the indirect effect, the estimated coefficient of green innovation by leading enterprises on enterprises in neighboring regions is significantly negative, indicating that green innovation by leading enterprises has a "siphoning effect", forming resource clusters by attracting innovation resources such as human and capital from other regions. Thus, negatively affecting green innovation by enterprises in other parts. However, this negative effect weakens as the geographical distance between areas increases. This may be because geographical distance increases the cost of resource flows and discourages the flow of resources across regions. This finding supports the previous research Hypothesis 3.

The above spatial econometric model systematically analyzes the spatial spillover effect of customer discourse power on the green innovation of leading enterprises. However, including more enterprises may bring the complexity of multi-factor association, thus increasing the difficulty of research. Therefore, this paper simplifies the spatial measurement. Future research will comprehensively consider the interaction of multiple factors. On the other hand, the green innovation of leading enterprises is a process of dynamic change.

## Research conclusions and implications

### Conclusions

Improving the green innovation capability of enterprises is an essential foundation for comprehensively promoting the national "double carbon" strategy and achieving high-quality development. The following are the paper’s primary conclusions: (1) There is an inverted "U" relationship between customer discourse power and the green innovation of leading companies. Additionally, the impact of customer discourse power on leading firms’ green innovation is amplified when companies are technology-intensive, have a high degree of industry specialization, or are situated in regions with a high level of marketization. (2) Further mechanism of action suggests that customer affects firms’ financing constraints, affecting the green innovation of leading firms. (3) In addition, the improvement of green innovation of leading firms helps to promote green innovation of non-leading firms in the local area, thus realizing the joint advancement of both. Although there is a "siphoning effect" on the innovation resources of businesses in non-neighboring areas, it also aids businesses in neighboring areas to raise their level of green innovation.

We combine social network theory with a new economic geography research perspective. Social network theory emphasizes the importance of network relationships and ties. On the other hand, the new economic geography research perspective suggests that the mobility of factors and the diffusion of information technology can increase the dependence between firms. This study places both into the same logical, analytical framework to explore the green governance relationship between customers and supplier-leading firms. First, we provide a detailed empirical analysis of social network theory, showing that customers play a vital role in green innovation by leading firms, i.e., leading firms can obtain innovation resources from customer firms and also be restricted from green innovation activities by excessive discourse power of customer firms. This finding is generally consistent with existing studies, which indicate that the partnership between the company and the customer company will be a crucial point in green innovation, and the strength of the bond will significantly affect the firm’s willingness to innovate and access innovation resources. Second, we provide empirical evidence for the research related to green innovation of firms. It shows that firm-customer interaction will influence firm green innovation. Moreover, the green innovation activities of leading enterprises also help empower SMEs to green innovation, help to play the role of radiation of leading enterprises and provide green innovation support to neighboring enterprises.

### Implications of the study and further research

#### Local governments

To support the ingrained growth of leading firms and increase the innovation spillover impact of leading local enterprises, local governments should design an effective and cooperative innovation framework. On the one hand, policymakers should use laws to control the industrial chain’s development, and they ought to promote the power of leading businesses to innovate sustainably through fair policy tilts toward innovation, innovation subsidies, and other measures. On the other hand, local governments need to challenge the notion of "local" innovation, encourage the establishment of regional innovation networks and city clusters, and bolster the spatial interaction effect of green innovation.

#### Leading enterprises

Prominent businesses should take the arbitrary lead in environmentally friendly innovation. Leading businesses in the supply chain with a high concentration of customers ought to connect with financial institutions, create external channels to reduce funding barriers through involvement in pertinent financial institutions and ensure that green innovation initiatives continue. Leading companies should also focus on the synergies within the industry chain, develop appropriate mechanisms for interacting with small and medium-sized businesses in the chain, and fully utilize their role as the leading company in driving innovation by "leading small enterprises with large enterprises" through industrial collaboration and geographic cooperation.

#### Non-leading enterprises

To prevent impeding the process of other regions’ green empowerment by hastily developing local green innovation, enterprises should identify their network location and use complementary technology, capital, and other related resources.

We provide important evidence on the relationship between customer discourse power and green innovation in leading firms. However, there are still some limitations. In particular, this study only focuses on manufacturing and other industries in. In particular, this study only focuses on the effect of customer discourse power of leading enterprises on green innovation in industries such as manufacturing, ignoring the relationship between the two and the existence of special mechanisms of action in industries such as finance and insurance. In the future, with the continuous promotion of the "double carbon" goal and the continuous improvement of China’s green financial system, the relationship between customers of leading enterprises and green innovation in the financial industry can be explored in depth. In addition, future research can be conducted to segment the categories of green innovation.

## Supporting information

S1 Data (DTA)
